# Inhaled Corticosteroids Increase the Risk of Pneumonia in Patients With Chronic Obstructive Pulmonary Disease: A Nationwide Cohort Study: Erratum

**DOI:** 10.1097/MD.0000000000008579

**Published:** 2017-11-03

In the article, “Inhaled Corticosteroids Increase the Risk of Pneumonia in Patients With Chronic Obstructive Pulmonary Disease: A Nationwide Cohort Study”,^[1]^ which appeared in Volume 94, Issue 42 of *Medicine*, Dr. Chih-Hsin Lee's affiliation should have appeared as Division of Pulmonary Medicine, Department of Internal Medicine, Wanfang Hospital and Division of Pulmonary Medicine, Department of Internal Medicine, School of Medicine, College of Medicine, Taipei Medical University, Taipei, Taiwan.
